# Exploring the Impact of E-cigarettes on Cardiovascular Health: Insights from Preclinical and Clinical Studies

**DOI:** 10.1007/s12012-025-10046-y

**Published:** 2025-08-19

**Authors:** Tijana Simovic, Chloe L. Matheson, Marisa Colon, Caroline O. Cobb, Judith Voynow, Youngdeok Kim, Patrick Nana-Sinkam, Ryan Garten, Paula Rodriguez-Miguelez

**Affiliations:** 1 Department of Kinesiology and Health Sciences, Virginia Commonwealth University, 817 West Franklin Street, Richmond, VA 23284 USA; 2 Department of Psychology, Virginia Commonwealth University, Richmond, VA USA; 3 Division of Pediatric Pulmonology, Virginia Commonwealth University, Richmond, VA USA; 4 Division of Pulmonary and Critical Care, Virginia Commonwealth University, Richmond, VA USA

**Keywords:** E-cigarettes, Tobacco products, Cardiovascular health, Cardiotoxicity

## Abstract

Electronic cigarettes are popular tobacco products that heat e-liquid into an aerosol for inhalation. Since their introduction to the market, electronic cigarettes have been considered a safer alternative to combustible tobacco products. However, most of today’s users are adolescents and young adults naïve to tobacco products, who are drawn to e-cigarettes by appealing designs and targeted marketing, resulting in exposure to largely unknown short- and long-term health risks. While the cardiovascular effects of electronic cigarettes remain incompletely understood, there has been a growing concern surrounding their potential acute and chronic impact on cardiovascular health and disease risks. In this review, we examine the components of e-cigarettes and evaluate the cardiovascular effects of both acute and regular e-cigarette exposure, summarizing findings from existing preclinical and clinical studies to provide a comprehensive overview about the topic.

**Graphical Abstract:**

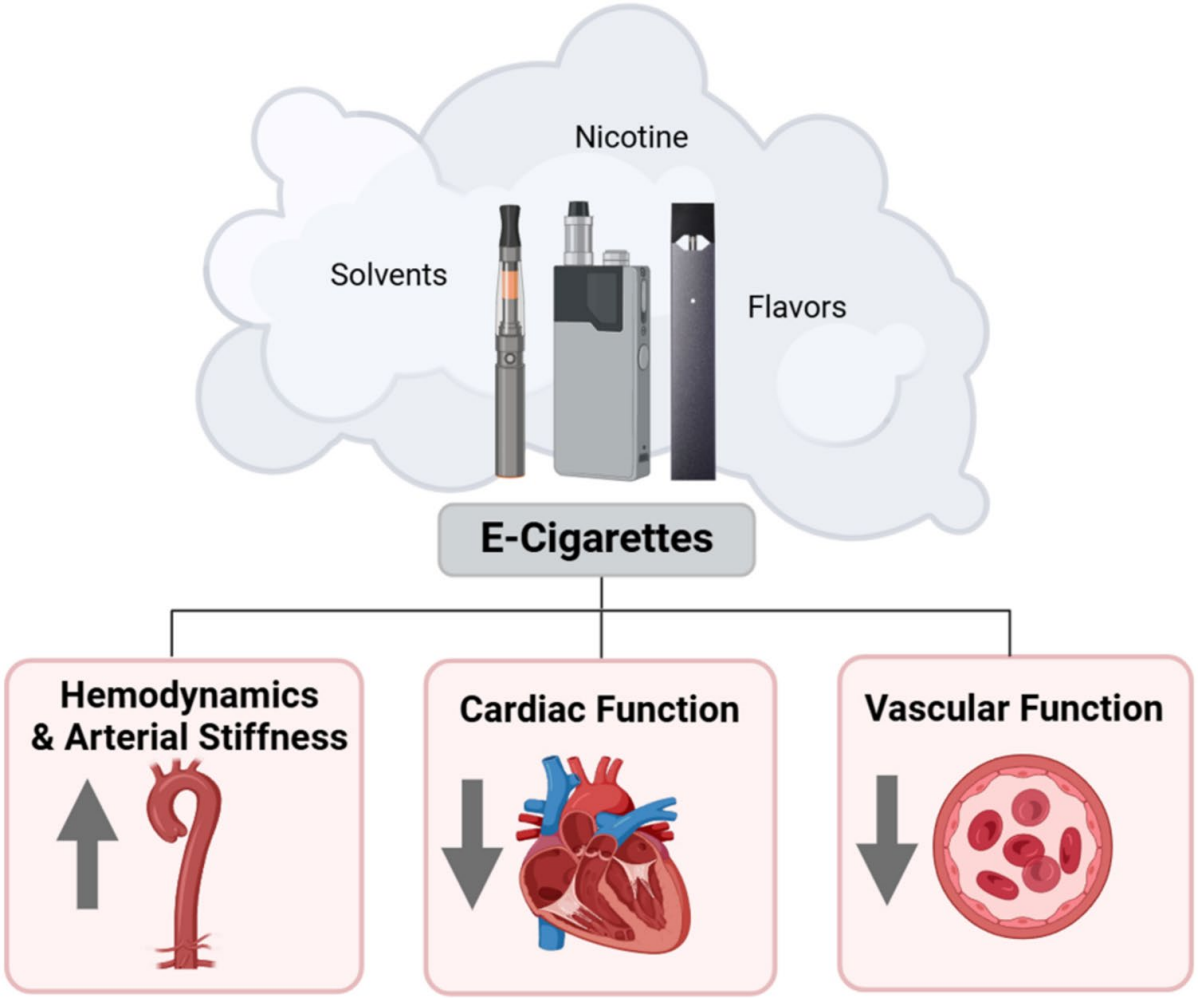

## Introduction

Electronic nicotine delivery systems (ENDS), commonly known as electronic (e-) cigarettes or vapes, are battery-powered devices that deliver aerosol, containing nicotine and other constituents such as propylene glycol, vegetable glycerin, and flavorings, classified by regulatory agencies as tobacco products [1]. Since their introduction to the U.S. marketplace in 2007, e-cigarettes have evolved into varying shapes, sizes, and wattages, becoming more compact and discreet, offering greater varieties of flavors, all while yielding aerosol containing high concentrations of nicotine [2, 3].

The rapid rise in their popularity is attributable to the perceived notion that e-cigarette products represent a healthier alternative to combustible tobacco products [4]. However, individuals who previously consumed combustible cigarettes and switched to e-cigarettes make up a segment of today’s adult users [5, 6]. Due to the wide variety of flavors and marketing tactics that seem to favor younger consumers [7], nowadays, e-cigarettes are frequently used by youth (9–17 year) [8] and young adults (18–24 year) [5], most of whom have never consumed combustible tobacco products [6, 9].

In recent years, several studies have raised concerns over possible adverse health effects associated with ENDS containing nicotine and the mixture of chemicals within the e-liquid vehicle [10]. With an increase in popularity among youth and young adults, concerns over the possible negative health effects have become necessary to investigate. Thus, in this comprehensive narrative review, we will provide an update on the current knowledge surrounding the effects of e-cigarettes on cardiovascular health.

## Electronic Cigarettes Compounds

ENDS are compact battery-powered devices that mimic combustible tobacco cigarettes with a modern design, producing an aerosol that the individual inhales. Over the last decade, variations of the original e-cigarette have continued to be produced and evolve as demand increased [11]. Most ENDS sold today have progressed from traditional box-shaped devices to sleeker, more compact single-use products. With a more attractive design, new generations of e-cigarettes have become increasingly popular, likely due to their user-friendly function, wide range of flavors, and ability to be used discreetly [7]. The majority of ENDS currently available on the market comprised a cartridge containing e-liquid composed of nicotine, flavoring, and varying levels of solvents, as well as a power source and heating element/coil used to aerosolize the e-liquid for consumption [12].

Initial ENDS products delivered lower concentrations of nicotine than combustible tobacco products [13]. However, a second generation of e-cigarettes evolved with functionality and design changes that allowed higher nicotine delivery, though still lower than combustible cigarettes [13]. Third-generation e-cigarettes, known as “mods”, offer consumers the possibility to modify device characteristics including power, allowing users to build custom modifications to the vaping devices, creating a more personalized experience [14]. To note, changes in wattage associated with some third-generation ENDS may increase nicotine delivery relative to other e-cigarette products [15]. Indeed, third-generation devices with lower nicotine concentrations and higher wattage resulted in greater nicotine plasma levels than second-generation products containing higher nicotine concentrations and lower wattage [15]. Currently, the fourth generation and most popular style of e-cigarettes, known as the pod-style, offers a variety of nicotine e-liquid concentrations that range from 0 to 5% [16], with varying wattage and resistance amounts [17].

### Nicotine

Nicotine is a well-known plant derivative with addictive properties found in tobacco products, including e-cigarettes. Nicotine is absorbed through the bloodstream, with the rate of absorption greatly depending on the pH of the nicotinic solution [18]. Consumption of nicotine has been linked to a variety of types of cellular dysfunction, including elevated oxidative stress [19]. Separately, previous literature has suggested that nicotine facilitates an increase in tumor progression [20], and similar cell proliferation associated with lung cancer [21]. Elevation of pulmonary proteolysis was found to be similar between users of ENDS and consumers of combustible tobacco products, as both groups had elevated neutrophil elastase levels, a protease released by neutrophils, compared to never smokers. In addition, nicotine was found to be the primary contributor to elevations in protease activity in isolated human neutrophils and macrophages, when compared to e-liquid solvents [22]. Furthermore, nicotine has been shown to induce endothelial damage and apoptosis [23], with other studies suggesting that nicotine consumption through ENDS promotes tissue remodeling, causing vascular dysfunction via damaged endothelium [24]. Though nicotine has been associated with negative health implications, the long-term effects of ENDS containing nicotine consumption are still presently unknown [25].

As vaping products and devices changed and evolved, various formulations of nicotine also emerged. The first generations of ENDS contained primarily freebase (unprotonated) nicotine, known to be alkaline, resulting in greater harshness and a less desirable experience upon inhalation [26]. Changes in the distribution of nicotine forms led to the introduction of products with a greater proportion of protonated nicotine, achieved by adding one or more acids to a nicotine base, forming a nicotine salt. ENDS liquids containing nicotine salts allow for a higher amount of nicotine to be absorbed with a lower amount of irritation to the user’s airways [27]. The introduction of nicotine salts offered a similar experience to combustible cigarettes while minimizing the negative perception or sensation associated with combustible tobacco products [28] and creating a more “ideal” experience for users [2]. To note, the health impact of the use of nicotine salts is still unknown. Early reports supported that ENDS liquids consisting of nicotine salts increased plasma nicotine levels from 35 to 72% when compared to first-generation e-cigarettes containing freebase nicotine [29]. Further research is necessary to understand the full health effects of nicotine salts as well as the increase in plasma nicotine levels associated with current market products.

### Solvents

Apart from nicotine, e-liquids contain a variety of diverse chemical compounds. The vast majority of the volume that makes up commercial e-liquid contains propylene glycol (PG) and vegetable glycerin (VG). These known solvents act as a housing agent for flavors and nicotine [30]. Each e-liquid contains differing amounts of PG:VG mixture ratios, resulting in various experiences that fluctuate between brands [27]. PG and VG have been known to affect how nicotine is delivered in the bloodstream [31], the flavor profile of each e-liquid, as well as overall user experience [32].

Based on preliminary research, acute vaping of PG and VG with or without nicotine at high wattage (60W), promoted airway epithelial injury in young users of combustible tobacco products [33]. Similarly, in vivo and in vitro data also support that acute exposure to aerosolized PG:VG increases Mucin-5AC (MUC5AC) protein levels [34], a major airway gel forming protein known for its role in preventing infections [35] and a biomarker for COPD severity [36]. A separate study found similar results suggesting an increase in MUC5AC protein levels in culture human bronchial epithelial cells exposed to PG:VG aerosols [37] as well as an increase gene expression for several inflammatory markers including cytokines interleukin-6, interleukin-8 and matrix metalloproteinase-9 (MMP-9), known to play a role in the pathogenesis of COPD [37]. In addition, the thermal decomposition of PG and VG is linked to the creation of several harmful chemical compounds, including acetone, acrolein, formaldehyde, acetaldehyde, and benzaldehyde [38]. Moreover, a high oxidative and cytotoxic environment has been observed after exposing endothelial cells to ENDS aerosol content, regardless of nicotine concentration [39]. Additional observations support that exposure to e-liquid induces not only oxidative stress but also decreases total antioxidant capacity, leading to even greater systemic oxidative stress [40]. Current knowledge supports that aerosols produced by e-cigarettes pass through the alveoli and pulmonary system, and then, disseminated through the circulatory system [41]. The results of the absorption of these aerosols could have several negative effects, including increased oxidative stress [42] and inflammatory cytokine production [43], which can damage the vascular endothelium [44]. Similarly, aerosol inhalation can result in enhanced airway resistance [45] and increased sympathetic nervous system activity [46], factors contributing to elevated risk for the development of cardiopulmonary disorders [10, 47].

In vitro studies have also shown that exposure to vaporized e-liquid containing flavor additives resulted in an increase in DNA damage, oxidative stress [48], and cell death [49] in epithelial cells. Similarly, e-cigarette exposure disrupted protein membrane expression and induced a glycolytic, proinflammatory state in macrophages [33]. Despite the risk of this consumption, measuring the potential exposure can be difficult due to the ever-changing nature of e-liquids and e-cigarette products.

### Flavor Additives

E-liquids contain a variety of flavor additives to enhance the overall experience for ENDS users. In 2009, characterizing flavor additives in combustible tobacco products, excluding menthol, were officially banned [50]. However, it was not until 2016 that e-cigarettes and flavored e-cigarette e-liquids were defined as tobacco products, making them eligible for regulation under the FDA [51]. Later, in 2019, Massachusetts became the first state to ban the sale of all flavored e-cigarette liquids and tobacco products [52], with New Jersey [53], as well as New York, and Rhode Island banning all flavors outside of tobacco or menthol the following year [54]. In an effort to curb use among younger consumers, flavor additives in cartridge-based e-cigarettes (i.e., cinnamon and vanilla) were also banned in 2020 by the FDA [55]. More recently, in 2022, the state of California followed Massachusetts’ lead, becoming the second state to place major sales restrictions on flavor additives in most tobacco products, which included menthol [56]. However, ENDS are still available in a wide range of flavors across stores in the United States and online, making them readily accessible and enticing to younger consumers.

The knowledge related to flavor additives and their impact on cellular health is limited. Early observations supported that human monocytes exposed to specific flavoring chemicals such as cinnamaldehyde, vanillin, and pentane-dione, found in cinnamon, vanilla, and buttery flavor e-cigarettes, respectively, resulted in a significantly elevated proinflammatory profile [57, 58]. Similar results were also identified in lung epithelial cells exposed to various flavor additives such as acetoin (sweet and buttery), orthovanillin (vanilla), and cinnamaldehyde (cinnamon), which exhibited an increase in proinflammatory cytokines synthesis [59]. Endothelial cells exposed to various e-liquids containing flavor additives such as cinnamon and menthol also demonstrated disruptions of cell homeostasis [60], increasing reactive oxygen species, and endothelial dysfunction [61].

Cooling flavors such as mint or menthol are frequent additives in popular flavor combinations as they reduce perceived airway irritation, allowing for a more enjoyable smoking experience [62]. Menthol is often sold as an individual flavor found in all tobacco products. With the recent restrictions on other popular flavored e-liquids and vapes, there was a shift in consumption toward menthol-flavored [63] and synthetic cooling [64] products. Similar to other flavor additives, menthol has been shown to reduce vascular endothelial function by impairing nitric oxide bioavailability and increasing inflammation in aortic endothelial cells, with higher concentrations resulting in a comparable proinflammatory state as other flavors such as cinnamon and eugenol (clover) [60]. This increase in inflammation and oxidative stress associated with menthol is independent of nicotine and e-liquid vehicles [65]. Thus, recent policy proposals have also urged the inclusion of menthol in flavor bans [66], as new research has suggested negative health consequences of consuming menthol-flavored ENDS [65], similar to other flavors [60, 67].

Popular flavor additives have been shown to provoke cellular damage and increase inflammation, causing dysfunction in cellular mechanisms and pathways and resulting in potential long-term health consequences. Knowledge surrounding e-liquid flavors suggests additional cellular damage outside of other compounds already established in e-liquid, such as nicotine and solvents. Though some legislation has attempted to control the ever-expanding industry, additional knowledge regarding possible negative effects associated with flavor additives and how these additives increase ENDS consumption requires further research.

## Electronic Cigarettes, Hemodynamic Changes, and Arterial Stiffness

Combustible tobacco product consumption is a well-known contributor to a wide range of side effects impacting overall cardiac hemodynamics, including elevations in heart rate [68], blood pressure [69], and changes in arterial stiffness [70], all associated with cardiovascular disease development including hypertension, myocardial infarction, aortic aneurysm, atherosclerosis, stroke, or even greater mortality [71].

Data related to e-cigarette product use is limited, but early findings have described elevations in heart rate [72] even after a single exposure [24, 73]. To note, increases in heart rate have been comparable between e-cigarettes and combustible tobacco products immediately following consumption [41] and 30 min [74] after use. Moreover, heart rate was increased up to 120 min following exposure to ENDS with nicotine, even in frequent young users of combustible tobacco products [24]. Considering that prolonged alterations in heart rate are associated with an increased risk of cardiovascular diseases [75–77], these results emphasize the importance of evaluating the long-term impact of frequent e-cigarette usage on hemodynamic markers.

Similarly, blood pressure changes have been repeatedly identified as elevated after the use of combustible tobacco products, with acute alterations being observed even after exposure to low amounts of nicotine [78]. To note, some studies have suggested that individuals with a history of hypertension who used combustible tobacco products exhibited a notable reduction in systolic blood pressure (SBP) and diastolic blood pressure (DBP) when they switched to ENDS [79]. However, others have identified hemodynamic changes specifically associated with the use of ENDS containing nicotine [30], with users of ENDS presenting similar SBP and DBP to those who use combustible tobacco products [80, 81]. Additionally, e-liquid without nicotine may also drive hemodynamic alterations, as increases in SBP and DBP post-consumption were also identified after the use of nicotine-free ENDS [24].

Multiple observations have also supported early impairments in arterial stiffness following acute consumption of ENDS containing nicotine [24, 30, 80], even after very short periods of usage [82]. Note that changes in arterial stiffness were still evident even twenty minutes post-usage [80, 82], suggesting the potential for more prolonged changes. Other studies have identified comparable rises in arterial stiffness between combustible tobacco products and ENDS containing nicotine as an observable rise in arterial stiffness, which was noted for 15 min after using either product [73]. These findings support the idea that the use of electronic cigarettes may contribute to increased arterial stiffness to a comparable level to combustible tobacco products [73, 80]. It is also important to note that the use of nicotine-free e-cigarettes may also elicit similar changes in arterial stiffness comparable to use of ENDS containing nicotine and combustible tobacco cigarettes [41] as well as heated tobacco [83]. These results emphasize that other ENDS compounds, independently of nicotine, may additionally contribute to these detrimental cardiovascular effects [41, 83].

Due to the novelty of e-cigarettes and the young demographic of individuals who use FENDS, information related to the chronic effects of these products on humans remains limited. Preclinical studies have allowed a glimpse into the health effects of these products and the potential lasting negative impact. Rodent longitudinal models suggest that after eight months of exposure to e-cigarettes containing nicotine, significant differences in arterial stiffness were observed, similar to mice exposed to combustible tobacco product smoke [84]. Separately, rodents exposed to either combustible tobacco cigarettes or ENDS containing nicotine had comparable increases in SBP and DBP after eight weeks, with changes observable up to sixty weeks [85]. In humans, regular e-cigarette usage may suggest similar hemodynamic changes (heart rate and blood pressure) and arterial stiffness between users of e-cigarettes (naïve and unknown prior tobacco use) and non-users [86, 87]. These results were not surprising considering the participants’ young age, relatively short e-cigarette use (~ 4 years), and the extensive time it can take for impairments to develop. Whether these acute changes may translate to chronic impairments with continuous use is currently unknown. Further research is required to understand how the use of these products might negatively impact the cardiovascular system.

## Electronic Cigarettes and Cardiac Function

Chronic elevations in heart rate, blood pressure, and arterial stiffness have been shown to increase the risk of adverse cardiac remodeling [88, 89]. Despite the preliminary evidence presented earlier, the potential adverse effects of aerosols, nicotine, and other ENDS components on cardiac function are yet to be evaluated.

Information from preclinical studies infers adverse effects of e-cigarette vapor on cardiac function and structure. For example, observations in a mouse model suggest that acute exposure to e-cigarettes promoted arrhythmias and impaired ventricular repolarization [90]. Regarding chronic exposure, cases of cardiac fibrosis [91], increased cardiac inflammation [92], and enlarged left ventricle [84] have been identified in preclinical models exposed to e-cigarette aerosol for three to eight months. Additional evidence also supports increases in cardiac inflammation, oxidative stress, and fibrosis after prolonged exposure to electronic cigarettes, which was comparable to damage induced by combustible tobacco products [93]. In addition, reductions in systolic function [94], ejection fraction [95], increased systemic arterial resistance [96], as well as structural abnormalities [95, 96] have all been observed in rodent models exposed to aerosol with nicotine. It is worth noting that changes in ejection fraction have been associated with nicotine content since mice exposed to aerosols without nicotine did not exhibit those impairments [94]. Interestingly, preliminary evidence has also inferred that cardiac damage may be impacting males faster than females [97], primarily due to sex-dependent differences in nicotine metabolism [97]. A recent study identified deleterious changes in the left ventricular structure leading to cardiovascular dysfunction and increased systemic arterial resistance [96].

Considering the novelty of ENDS products, minimal information is available regarding e-cigarette usage and cardiac function in humans. Preliminary evidence supports that acute use to combustible tobacco products, but not second-generation ENDS aerosol, induced left ventricular myocardial relaxation [98]. Similarly, no difference in baseline cardiac electrical activity was identified between regular users of electronic cigarettes, regular users of combustible tobacco, and non-users [99]. Other reports suggest that acute exposure to combustible tobacco products and electronic cigarettes with nicotine [100], but not without nicotine [99], caused abnormal ventricular repolarization. Additionally, increases in stroke volume have been observed within five minutes of usage of a fourth-generation ENDS, similar to the observed responses after the use of a combustible tobacco product [82].

Results from preclinical and single-exposure clinical studies imply negative impacts of ENDS usage on myocardial structure, function, and electrophysiology, all related to cardiovascular disease development [101–103]. Recently, another study associated regular ENDS use with lower cardiorespiratory fitness [104], an independent predictor of cardiovascular disease‐related mortality [105]. Due to the novelty of these products and the lack of longitudinal data, additional research is needed to further understand the long-term effects of ENDS, aerosols, and nicotine on cardiac function.

## Electronic Cigarettes and Vascular Function

The link between combustible tobacco product usage, vascular endothelial dysfunction, and subsequent cardiovascular disease development is well documented [106]. Combustible tobacco product-induced vascular damage has been related to increases in oxidative stress, inflammation, altered hemodynamics, and autonomic dysregulations [107, 108]. However, the available information evaluating the impact of ENDS use on vascular health and potential mechanisms of action is limited.

Initial *in vivo* investigations using different animal models have explored the impact of acute exposure to electronic cigarette vapor on cardiovascular health [109–112]. Reports suggest that exposure to e-liquid aerosol causes rapid impairment in endothelial-dependent vascular dilation in rats, regardless of specific e-cigarette type or nicotine content [109, 110]. An additional study has explored which component of e-cigarette liquid (independent of nicotine and/or flavorings) may be contributing to these adverse vascular outcomes [112]. Interestingly, the exposure to PG-VG mixture and formaldehyde, but not acetaldehyde, led to endothelial dysfunction in female mice only [112], indicating the potential harm of frequent e-liquid components, as well as possible sex-dependent sensitivity to different electronic cigarette constituents [112]. A recent study further supported these findings by associating e-cigarette-induced endothelial impairment with formaldehyde exposure [113].

Recent studies have also provided further information on the effects of acute ENDS usage in adults, supporting that a single exposure to these products reduced vascular function [86] and an associated increase in oxidative damage [114, 115]. To note, similar reductions in endothelial-dependent dilation have been identified after a single exposure to combustible tobacco products or e-cigarettes [115], although contradictory results are also available, supporting that acute exposure to neither combustible tobacco cigarettes nor ENDS had an effect on vascular function [87]. Additional information supported that combustible tobacco products impaired vascular dilation while exposure to ENDS did not [116]. In this study, second-generation e-cigarettes were used, eliciting similar circulating nicotine concentrations to combustible tobacco, suggesting that other non-nicotine elements may be the primary cause of vascular dysfunction [116]. Opposite results have also been described with an acute use of nicotine-containing e-liquid decreasing endothelial-dependent vasodilation, whereas no changes were observed after use of nicotine-free e-liquid [24]. Indeed, an increase in oxidative stress was identified only after the inhalation of nicotine-containing e-liquids, highlighting the role of nicotine in the development of ENDS-induced vascular dysfunction [24]. However, a more recent study has described impaired endothelial function even after a single nicotine-free ENDS session that was also accompanied by increased inflammatory mediators (i.e., serum CRP, plasma HMGB1) and oxidative stress [117].

Regarding long-term exposure, limited information is available exploring the impact of these products on vascular health. In animal models, chronic exposure to e-cigarette aerosols reduced vascular function [84–86, 118, 119]. Specifically, exposure to short (five days [86]), medium (eight months [85]), and longer (fifteen months [118]) e-cigarette aerosol diminished endothelial function, accompanied by decreased intravascular nitric oxide bioavailability and elevated oxidative stress [118]. Similar findings confirmed that chronic exposure to ENDS vapor (four to fifteen months) with ranging nicotine concentrations (0–24 mg/ml) led to vascular dysfunction impacting both endothelial-dependent and endothelial-independent mechanisms [85]. Notably, endothelial dysfunction was developed in animals exposed to both nicotine and non-nicotine e-cigarettes, although those exposed to nicotine developed it at a faster rate [85]. Additional evidence also identified impaired endothelial-dependent dilation in mice exposed to nicotine-free and flavor-free ENDS aerosol [119], supporting the potential vascular toxicity of the primary e-liquid components, PG and VG.

Studies investigating the long-term effects of ENDS use on vascular function in humans are sparse. Initial observations described no differences in endothelial-dependent dilation between habitual users of combustible tobacco products, users of electronic cigarettes, dual users, and non-users [116, 120], although lower endothelial cell nitric oxide production has been identified in users of ENDS and users of combustible tobacco products when compared to non-users [120]. These findings are surprising considering the well-documented damage of combustible tobacco usage [121, 122] or even secondhand smoke exposure [123] on vascular function. The authors speculated that their results were related to the light usage (i.e., < 20 pack-years), young age (i.e., 29 yo,) and relatively healthy lifestyles (i.e., physical activity, abstinent from recreational drug use) of both combustible tobacco and ENDS groups [116, 120]. Similarly, recent studies observed comparable vascular function [87, 124] and systemic inflammatory markers [124] between young exclusive users of e-cigarettes and non-users. On the other hand, contradictory results have also been described, with regular users of ENDS exhibiting lower endothelial-dependent vascular dilation and diminished nitric oxide production when compared to non-users, with concomitant increased inflammatory biomarkers [125]. It is important to note that the observed difference between users of e-cigarettes and non-users was ~ 5%, and decreases of 1% in vascular dilation through the flow-mediated dilation test have been associated with an 8 to 11% greater risk for cardiovascular events [126]. Thus, the results from this study infer that those who are regular users of ENDS may exhibit ~ 40% or greater risk of future cardiovascular events when compared to non-users. Similar results were also observed in other vascular beds, including the microvasculature [127, 128], representing early changes in the cardiovascular system [129]. Indeed, two separate cohorts of young users of ENDS described reduced endothelium-dependent vasodilation and decreased nitric oxide bioavailability when compared to non-users [127, 128]. Additionally, individuals who used e-cigarettes for longer than three years not only exhibit reduced microvascular function but also diminished macrovascular dysfunction, implying that those who use the product for a longer time are at risk of systemic vascular effects [128].

Some studies have also investigated the potential vascular changes when users of combustible tobacco switched to e-cigarettes, both with and without nicotine. In both cases, the switch from combustible tobacco cigarettes to ENDS exerted similar improvements in endothelial function after one [130] or six [131] months. Results support increased nitric oxide bioavailability and improved vascular smooth muscle function after switching products [131]. Similar results were also observed in a group of current consumers of combustible tobacco products who exhibited a decrease in oxidative stress four months after switching to ENDS use, even when nicotine was still present [132]. Thus, the current evidence infers improvements in vascular function in this population, however, it is currently unknown whether these effects will remain following years of e-cigarette use.

In summary, preclinical data support negative vascular consequences following exposure to e-cigarette aerosol. In humans, some studies investigating acute and chronic exposure to e-cigarettes have identified adverse effects of ENDS use on vascular health, particularly in those with no history of prior combustible tobacco. However, other studies did not observe such effects, raising the concern that potentially longer follow-ups and/or use time are needed to assess the long-term impact of these products on vascular function. On the other hand, potential vascular improvements have been described in those who switched from consuming combustible tobacco products to electronic cigarettes. While oxidative stress and endothelial dysfunction seem to be common factors, the specific mechanisms driving ENDS-induced vascular dysfunction remain unknown and require further investigation.

## Future Perspectives

Despite the growing evidence suggesting potential cardiovascular harm associated with e-cigarette use, significant gaps remain. Among the greatest challenges when investigating the long-term health effects of e-cigarette use lies in the variability in accessible products and user behaviors. Around 20,000 different e-cigarette products are available for purchase worldwide [133], varying from product brands to flavors, e-liquid composition, and nicotine concentration, all of which may lead to distinct physiological responses. In addition, some users may choose to use a single brand of products while others might explore different brands and/or flavors [134], further complicating analysis of the ramifications of use. Moreover, user behaviors such as frequency of use, puffing patterns, motivation to use, and e-cigarette dependence may also result in individual variations in physiological responses. There remains an unmet need and lack of a standardized approach to comprehensively evaluate these variables, especially when considering the rapidly changing tobacco product market.

While this review summarizes the current evidence from preclinical and clinical studies, most of the existing findings are based on animal models or short-term use human studies, which may not fully reflect the consequences of regular use in humans. In addition, the preliminary long-term work in sole users of e-cigarettes identified early markers of cardiovascular disease development that were limited to a single timepoint during early adoption of e-cigarette use. It is unknown whether the prolonged use of e-cigarettes will result in continued adverse cardiovascular effects and/or if the identified mechanisms will be impacted in the same pattern over a longer period of time. Thus, longitudinal studies are essential to ascertain whether deleterious health effects will persist or evolve.

Similarly, the limited number of studies conducted in long-term sole users of e-cigarettes have been completed in regional cohorts, where environmental factors, as well as the laws and restrictions on e-cigarette products, can vary. Therefore, investigating the same research questions in larger cohorts geographically located in different regions may add additional insights. To note, a limited number of epidemiological reports evaluated the role of e-cigarette use in cardiovascular disease development. A few studies associated the use of these products with a higher risk for hypertension [135], stroke [136], myocardial infarction, and arrhythmias [137].

Moreover, studies investigating the cardiovascular effects following e-cigarette cessation are needed to determine whether the discontinuation results in improvements in cardiovascular health or lifelong consequences. Future interventional studies including pharmacological, lifestyle, and e-cigarette reduction strategies that will target physiological mechanisms and develop therapeutic approaches are also essential.

The question of whether e-cigarettes are healthier alternatives to combustible tobacco products remains a controversial one with an answer that has yet to be elucidated. The current evidence suggests improvements in cardiovascular health among long-term users of combustible tobacco products who switched to e-cigarettes; however, due to the pre-existing damage from prior combustible tobacco product use, it is difficult to isolate the cardiovascular effects of e-cigarette use in this population. Similarly, short follow-ups (i.e., 3–6 months) may not be sufficient to observe potential meaningful improvements or further assess harm attributable to only e-cigarette use. Therefore, future studies should investigate the long-term effect of e-cigarettes compared to combustible tobacco products on cardiovascular health among different populations, including various ages and disease states.

**Fig. 1:**
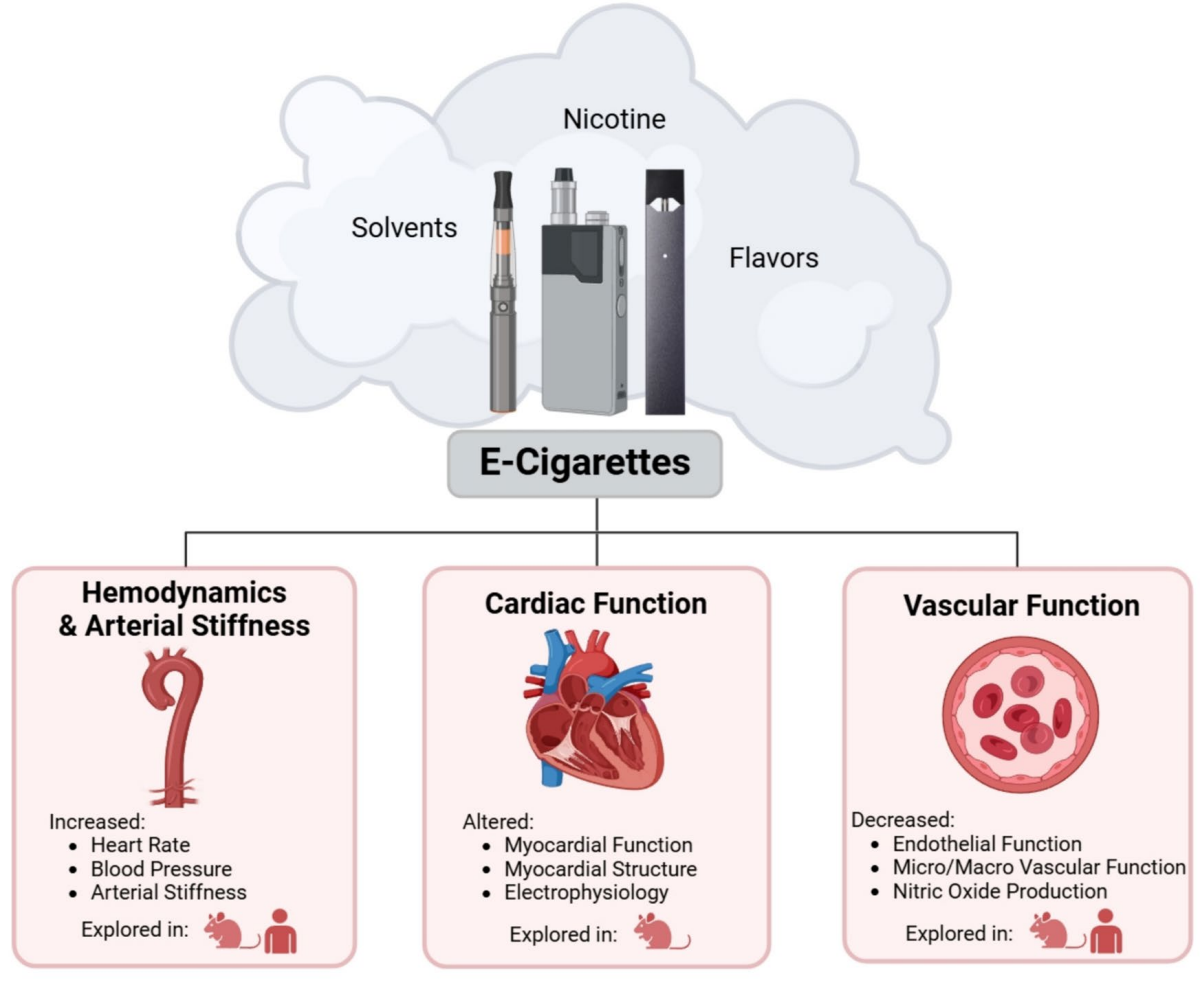
The effect of e-cigarette usage on cardiovascular health outcomes. A schematic overview of the cardiovascular effects associated with e-cigarette use. This figure highlights the adverse impact on hemodynamics and arterial stiffness, cardiac function, and vascular function, observed in both clinical and preclinical models, which contribute to heightened cardiovascular disease risk. Figure created with BioRender and published with permission

Beyond the potential negative effects of ENDS usage on cardiovascular function, a significant gap in understanding how e-cigarette use may affect other major organ systems remains. Studies conducted in rodents identified that exposure to e-cigarette aerosols adversely affected the bone structure and density [138] as well as endocrine function in both male and female reproductive systems [139]. In addition, nicotine has been documented to have a harmful influence on the central nervous system, with research suggesting e-cigarette aerosols delaying brain development and altering cognition [140], raising concerns given the high prevalence of ENDS use among youth and young adults. Moreover, evidence indicates that e-cigarettes weaken the immune system, as exposure has been shown to induce altered immune response and homeostasis [141, 142] with significant disruptions specifically associated with fourth-generation e-cigarettes [143]. While early reports have also described a connection between acute e-cigarette use and biomarkers linked to elevated cancer risk, there is limited evidence at this point to suggest a direct causal association between ENDS usage and cancer development [144]. In summary, the major health complications of continued long-term e-cigarette usage are still mostly unknown and comprehensive and longitudinal studies are needed to better understand the full spectrum of potential adverse health risks associated with regular e-cigarette use (Fig. 1).

## Conclusions

Over recent years, e-cigarettes have gained popularity as a potentially less harmful alternative to combustible tobacco products. Preclinical studies have identified adverse cardiovascular effects associated with both acute and chronic exposure to e-cigarette aerosol. While the body of clinical research remains limited, emerging evidence indicates similar deleterious cardiovascular outcomes, particularly among young adults without a prior history of combustible tobacco product use—currently the largest demographic of individuals who use ENDS. Of note, some studies have reported improvements in cardiovascular health among individuals who transitioned from long-term combustible tobacco product use to e-cigarettes. Frequent components of these products, including nicotine, flavoring agents, and solvents, have each been implicated in adverse cardiovascular effects, although the precise mechanisms underlying these outcomes remain poorly understood. Further research is essential to elucidate these mechanisms and assess the broader implications of e-cigarette usage on cardiovascular health.

## Data Availability

No datasets were generated or analyzed during the current study.
